# Brain-Derived Neurotrophic Factor Val66Met Gene Polymorphism Impacts on Migraine Susceptibility: A Meta-analysis of Case–Control Studies

**DOI:** 10.3389/fneur.2017.00159

**Published:** 2017-05-01

**Authors:** Salvatore Terrazzino, Sarah Cargnin, Michele Viana, Grazia Sances, Cristina Tassorelli

**Affiliations:** ^1^Department of Pharmaceutical Sciences and Interdepartmental Research Center of Pharmacogenetics and Pharmacogenomics (CRIFF), University of Piemonte Orientale “A. Avogadro”, Novara, Italy; ^2^Headache Science Center, C. Mondino National Neurological Institute, Pavia, Italy; ^3^Department of Brain and Behavioral Sciences, University of Pavia, Pavia, Italy

**Keywords:** migraine, susceptibility, brain-derived neurotrophic factor, single-nucleotide polymorphism, meta-analysis

## Abstract

Inconclusive results have been reported in studies investigating the association between the brain-derived neurotrophic factor (BDNF) rs6265 polymorphism and migraine. In the present study, we conducted a systematic review and meta-analysis on the published data in order to quantitatively estimate the relationship between rs6265 and migraine susceptibility. A comprehensive search was performed through PubMed, Web of Knowledge, and Cochrane databases up to October 2016. The pooled odds ratio (OR) with the corresponding 95% confidence interval (CI) was calculated to estimate the strength of the association with rs6265 under an additive, dominant, or recessive model of inheritance. A total of five studies including 1,442 cases and 1,880 controls were identified for the meta-analysis. The pooled data showed an increased risk of migraine for the allelic (OR: 1.17, 95% CI: 1.03–1.34, *p* = 0.014) or the dominant model of rs6265 (OR: 1.22, 95% CI: 1.05–1.41, *p* = 0.011). Statistical significance of rs6265 was lost when one single study was excluded from the analysis (dominant OR: 1.17, 95% CI: 1.00–1.38, *p* = 0.054; allelic OR: 1.14, 95% CI: 0.99–1.31, *p* = 0.067), suggesting lack of robustness of pooled estimates. When stratified by migraine type, a similar trend of association was detected with both MA and MO, but a statistically significant association of rs6265 was reached only with the MA subtype in the dominant model (OR: 1.22, 95% CI: 1.00–1.47, *p* = 0.047). The present meta-analysis supports that BDNF rs6265 may act as a genetic susceptibility factor for migraine. Nevertheless, large-scale studies are required to confirm our findings and to assess potential modifiers of the relationship between rs6265 and migraine.

## Introduction

Migraine is a disabling neurovascular disease affecting more than 10% of worldwide population, with a female-to-male prevalence ratio of about 3:1 (1). Two major clinical subtypes of migraine have been classified by the International Headache Society (IHS): migraine without aura and migraine with aura (2). The more common type, migraine without aura (MO), is characterized by episodes of moderate to severe headache that is mostly unilateral, throbbing, and aggravated by routine physical activity. The headache pain is accompanied by other symptoms, such as nausea, vomiting, photophobia, and phonophobia (3). In migraine with aura (MA), attacks are preceded by transient focal neurological symptoms, mostly visual, less frequently somatosensory or dysphasic (4). Migraine is recognized as a multifactorial polygenic disorder involving a complex interaction between genetic and environmental factors (5). However, the exact etiology and the underlying pathological mechanisms are still to be completely understood (6).

The brain-derived neurotrophic factor (BDNF), a neurotrophin involved in synaptic plasticity and survival of neurons, has been reported to play also a role in the modulation of pain signaling and in central sensitization (7, 8). The effects of BDNF within the nociceptive system are manifolds and dose dependent, with low doses causing hyperalgesia, whereas higher doses lead to analgesia, an effect that might be induced by the activation of different intracellular pathways (9). Recently, a role of BDNF has been suggested in migraine pain, due to its interaction with calcitonin gene-related peptide (CGRP), a key vasodilating neuropeptide implicated in migraine pathogenesis. Indeed, BDNF is co-expressed with CGRP in trigeminal ganglion neurons (TGNs) (10) and CGRP induces BDNF release from TGNs, an effect that is reversible in the presence of a CGRP receptor antagonist (11). In addition, serum levels of BDNF were found increased in migraineurs compared to healthy controls (12) or during migraine attacks compared to pain-free periods (12, 13).

The BDNF gene encodes for a precursor peptide (pre-pro-BDNF), which is subsequently cleaved to generate the precursor of BDNF (proBDNF) and mature BDNF (14). Each of these BDNF forms exerts opposing effects on cell apoptosis, long-term depression, and synaptic plasticity through two different transmembrane receptor signalings (15). The most extensively studied variant of the BDNF gene is rs6265, also called Val66Met or G196A, a single-nucleotide polymorphism (SNP) resulting in a valine to methionine substitution at codon 66 in the pro-region of BDNF. This polymorphism affects intracellular packaging of pro-BDNF, its axonal transport and, in turn, activity-dependent secretion of BDNF at the synapse (16, 17). A number of candidate gene studies have focused on the role of BDNF rs6265 as a risk factor for migraine (18–21); however, the results remain inconclusive.

Candidate gene association studies have been criticized for a number of weaknesses including insufficient sample size of most studies or insufficient replication of the results (22). As these limitations can be partly overcome by combining data from relevant studies by meta-analytic methods (23), we herein conducted a systematic review and meta-analysis of published data to estimate the impact of BDNF rs6265 on migraine susceptibility.

## Materials and Methods

### Literature Search and Inclusion/Exclusion Criteria

The protocol for this review was published in the PROSPERO database of prospectively registered systematic reviews (registration ID: CRD42016049038) (24). PubMed, Web of Knowledge, and Cochrane Library databases were searched up to October 2016 using the Boolean combination of the following key terms: (BDNF OR brain-derived neurotrophic factor) AND migraine. Inclusion criteria were (1) case–control studies evaluating the association between BDNF rs6265 and migraine, (2) studies including healthy subjects as controls, and (3) studies containing genotype data. Exclusion criteria were (1) non-human studies, (2) case reports, (3) review articles and editorials, and (4) duplication of previous publications. There were no language restrictions. The retrieved studies were then read in their entirety to assess their appropriateness for inclusion in the meta-analysis. Corresponding authors of eligible studies were contacted *via* email when relevant data were not extractable from the published manuscript. One of them (25) generously provided data on rs6265 genotype distribution needed to calculate the Hardy–Weinberg equilibrium (HWE) and to compute odds ratios (ORs).

### Data Extraction and Quality Assessment

A standardized form was used for each study included in the qualitative analysis to collect the following information: last name of the first author, year of publication, study site, sample size for both cases (any migraine, MO and MA) and controls, sample characteristics (gender ratio, mean age, distribution of rs6265 genotypes), and detection method. Deviation of rs6265 from the HWE was calculated using the Pearson’s goodness-of-fit chi-square test implemented in the online Finetti’s program (available at: http://ihg.gsf.de/cgi-bin/hw/hwa1.pl). The quality of case–control studies was evaluated using a risk-of-bias score for genetic association studies, as adopted in a previous meta-analysis (26). Studies with a quality score equal or higher than the median score were considered of higher quality. All studies have been independently analyzed by two reviewers (Salvatore Terrazzino and Sarah Cargnin) and any discrepancies have been resolved through consensus.

### Statistical Analysis

For each study, the OR with the corresponding 95% confidence interval (CI) was calculated to estimate the strength of the association between BDNF rs6265 gene polymorphism and migraine. OR estimates were combined based on the allele (A vs G), dominant (i.e., GA + AA vs GG), and recessive (i.e., AA vs GA + GG) genetic contrast of rs6265 by using the random-effects (DerSimonian–Laird method) model, which takes into account any difference among studies even if there is no statistical heterogeneity (27). In case of lack of heterogeneity, the random-effects model coincides with the fixed-effect model (28). We estimated the between-study heterogeneity across all eligible comparisons by using the χ^2^–based Cochran’s *Q* statistic (significant for *p* < 0.10) (29). The *I*^2^ index (range 0–100%) was also reported, which quantifies heterogeneity irrespective of the number of included studies (30). Leave-one-out sensitive meta-analysis was performed to assess the contribution of each study to the pooled estimate by excluding individual results one at a time and recalculating the pooled OR estimates for the remaining results. Data analysis was performed with Open Meta-Analyst (available at: http://www.cebm.brown.edu/openmeta/) and ProMeta software Version 2 (INTERNOVI di Scarpellini Daniele s.a.s., Cesena FO, Italy), and significance of pooled estimates was set at *p* < 0.05. Publication bias was evaluated by the Egger’s test (31) using the ProMeta version 2 software. An Egger’s *p* value <0.10 was considered to indicate statistically significant publication bias. The sample size required for a replication study was determined by using the Quanto software (http://biostats.usc.edu/Quanto.html).

## Results

### Literature Search and Characteristics of Included Studies

**Figure 1:**
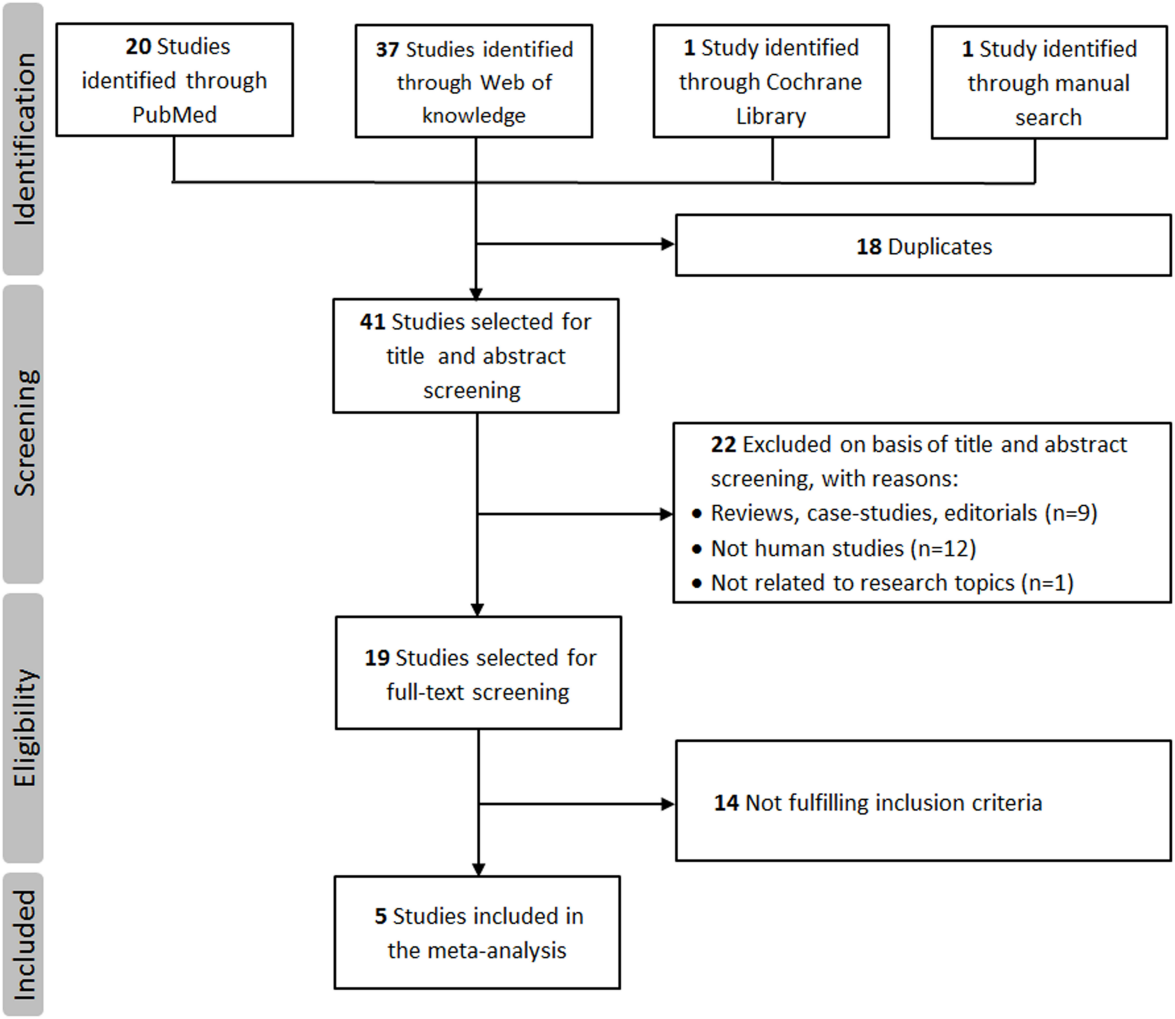
**Flowchart of the literature review process**.

A total of 59 studies were identified through a search of PubMed, Web of Knowledge, and Cochrane databases. After exclusion of 54 hits for not meeting the inclusion criteria, five studies with 1,442 cases and 1,880 controls were, therefore, included in the final meta-analysis (18–21, 25). The detailed flowchart of literature review process is shown in Figure 1. The main characteristics of studies included in the meta-analysis are shown in Table 1. In all studies, genotype distribution of rs6265 was in HWE both in cases and controls. The median quality score of studies included in the meta-analysis was 10 (range 8–11), indicating a low risk of bias. Methodological items for quality assessment and individual item scores of each included study are illustrated in Table 2. Three studies were classified as “higher” quality studies (19–21), while the remaining two studies were rated as “lower” quality studies (18, 25). Genotypic data for the BDNF gene rs6265 were available in four studies as subgroups of migraine with and without aura (18, 20, 21, 25).

**Table 1:** **Main characteristics of the studies included in the meta-analysis of the association between brain-derived neurotrophic factor rs6265 and migraine**.

Reference	Country	Eligible participants		Migraine type, MO/MA	Age, mean (SD) in years	Detection method	rs6265 genotypes	MAF	HWE	Quality score
		
		Cases (M/F)	Controls (M/F)		Cases/controls		Group: GG/GA/AA	Cases/controls	*p*-Value Cases/controls	
Marziniak et al. (18)	Germany	265 (43/222)	153 (43/110)	143/122	43.6 (13)/64.5 (9.4)	PCR-RFLP	Cases: 148/104/13	0.245/0.239	0.33/0.75	9
							MO: 80/57/6			
							MA: 68/47/7			
							Controls: 88/57/8			
Lemos et al. (19)	Portugal	188 (35/153)	287 (70/217)	111/77	36.1 (12.8)/36.4 (12.5)	Real-time PCR	Cases: 118/64/6	0.202/0.197	0.45/0.43	11
							Controls: 183/95/9			
Sutherland et al. (20)	Australia	857 (196/661)	857 (196/661)	212/645	43.7 (13.1)/44.8 (13.3)	PCR-RFLP	Cases: 396/193/23	0.195/0.172	0.93/0.87	10
							MO: 95/44/5			
							MA: 311/149/18			
							Controls: 544/224/24			
Coskun et al. (21)	Turkey	288 (137/151)	288 (133/155)	176/112	31.3 (10.3)/31.5 (8.9)	Real-time PCR	Cases: 196/84/8	0.174/0.132	0.78/0.99	10
							MO: 121/49/6			
							MA: 75/35/2			
							Controls: 217/66/5			
Azimova et al.^a^ (25)	Russia	90 (14/76)	363 (165/198)	74/15	40.0 (12.0)/37.2 (8.7)	PCR-RFLP	Cases: 58/29/2	0.185/0.140	0.46/0.97	8
							MO: 48/24/2			
							MA: 10/5/0			
							Controls: 266/87/7			

*HWE, Hardy–Weinberg equilibrium; MAF, minor allele frequency; MO, migraine without aura; MA, migraine with aura; PCR-RFLP, polymerase chain reaction-restriction fragment length polymorphism*.

*^a^Data have been provided by the corresponding author himself after being contacted*.

**Table 2:** **Quality assessment of studies included in the meta-analysis**.

Criteria	Score	Marziniak et al. (18)	Lemos et al. (19)	Sutherland et al. (20)	Coskun et al. (21)	Azimova et al. (25)
**Representativeness of cases**						
Consecutive/randomly selected from case population with clearly defined sampling frame	2	X	X	X	X	X
Consecutive/randomly selected from case population without clearly defined sampling frame or with extensive inclusion/exclusion criteria	1					
No method of selection described	0					
**Representativeness of controls**						
Controls were consecutive/randomly drawn from the same sampling frame as cases	2	X	X	X	X	X
Controls were consecutive/randomly drawn from a different sampling frame as cases	1					
Not described	0					
**Ascertainment of migraine**						
Clearly described objective criteria for diagnosis of migraine	2	X	X	X	X	X
Diagnosis of migraine by patient self-report or by patient history	1					
Not described	0					
**Quality control of genotyping methods**						
Clearly described a different genotyping assay to confirm the data	1		X			
Not described	0	X		X	X	X
**Hardy–Weinberg equilibrium**						
Hardy–Weinberg equilibrium in controls	2	X	X	X	X	
Hardy–Weinberg disequilibrium in controls	1					X
No checking for Hardy–Weinberg disequilibrium	0					
**Association assessment**						
Assess association between genotypes and migraine with appropriate statistics and adjustment for confounders	2		X	X	X	
Assess association between genotypes and migraine with appropriate statistics without adjustment for confounders	1	X				X
Inappropriate statistics used	0					
Total score	11	9	11	10	10	8

### Quantitative Synthesis

**Figure 2:**
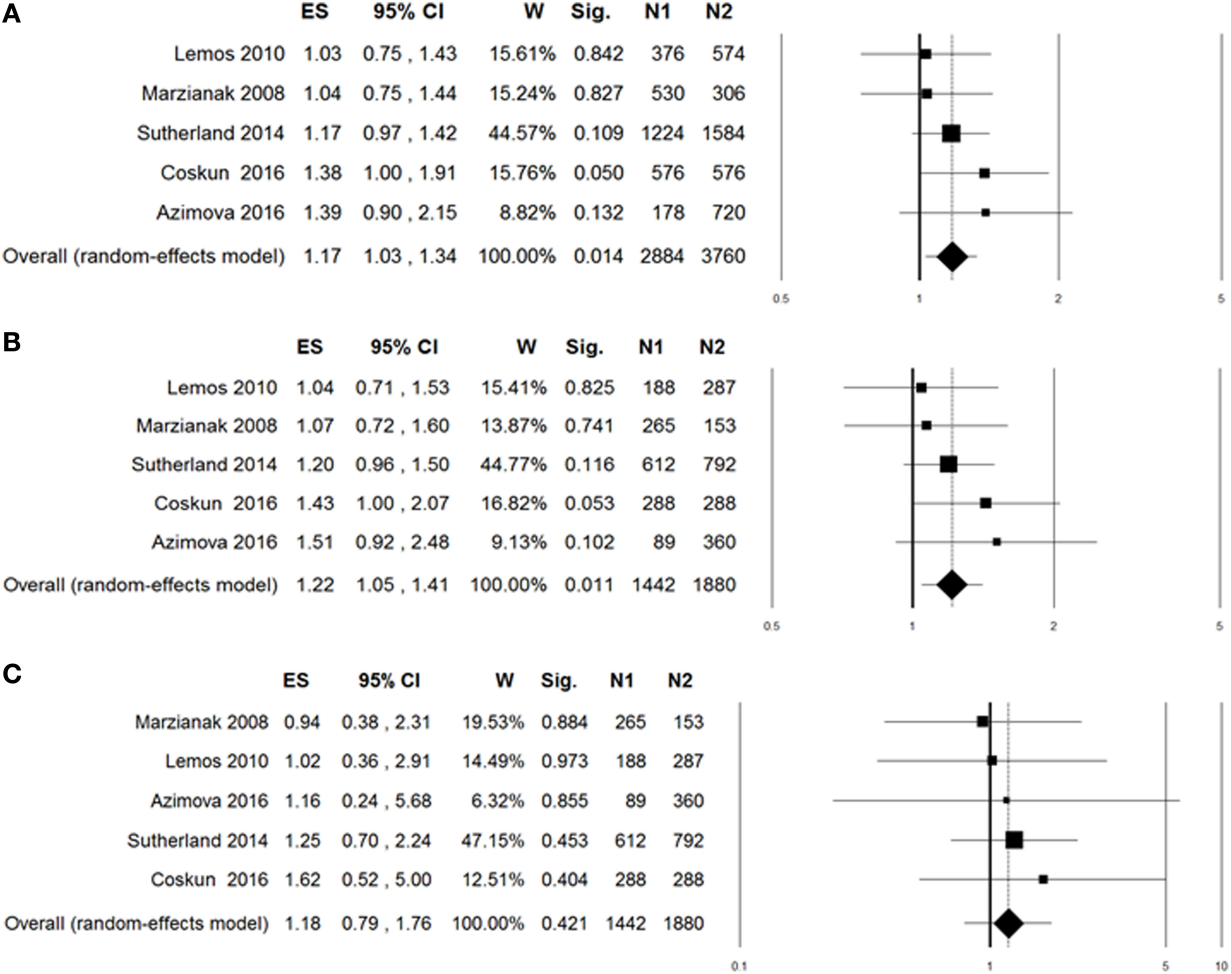
**Forest plots for the association between brain-derived neurotrophic factor rs6265 and migraine under the allelic (A), dominant (B), or recessive (C) genetic model of inheritance**. The summary odds ratio (OR) is represented by the diamond, where the center of the diamond indicates the OR and the ends of the diamond correspond to the 95% confidence interval. ES, effect size (OR); W, weight; Sig, statistical significance; N1, cases; N2, controls.

A summary of meta-analyses on the association between rs6265 and migraine risk is displayed in Table 3. The Q-statistic for the overall data indicated absence of study heterogeneity (allelic model: *p* = 0.60, *I*^2^ = 0%; dominant model: *p* = 0.63, *I*^2^ = 0%; recessive model: *p* = 0.95, *I*^2^ = 0%). The pooled analysis showed significant association in the allelic (OR: 1.17, 95% CI: 1.03–1.34, *p* = 0.014; Figure 2A) and dominant contrast of rs6265 (OR: 1.22, 95% CI: 1.05–1.41, *p* = 0.011; Figure 2B), but not in the recessive model (OR: 1.18, 95% CI: 0.79–1.76, *p* = 0.42; Figure 2C). No statistical evidence of publication bias was detected in the results (allelic model: Egger’s *p* value = 0.75; dominant model: Egger’s *p* value = 0.72; recessive model: Egger’s *p* value = 0.90). Nevertheless, given the low power of the Egger’s test to detect funnel plot asymmetry, especially when limited number of studies are included in the meta-analysis, small-study effects, or publication bias cannot be excluded.

**Table 3:** **Summary of meta-analyses for the association of brain-derived neurotrophic factor rs6265 with migraine risk**.

Group or subgroup	No. studies	Sample size	Odds ratio (95% confidence interval)	*p*	Heterogeneity		Egger’s *p*
		Cases/controls			*p* (*Q*-test)	*I*^2^(%)	
**Allelic model (A vs G)**							
Any migraine	5	2884/3760	1.17 (1.03–1.34)	**0.014**	0.60	0	0.75
MO	4	1074/3186	1.20 (0.99–1.44)	0.057	0.56	0	
MA	4	1454/3186	1.17 (0.99–1.38)	0.057	0.83	0	
Higher quality	3	2176/2734	1.18 (1.02–1.37)	**0.027**	0.46	0	
Lower quality	2	708/1026	1.16 (0.88–1.54)	0.30	0.29	12	
**Dominant model (GA + AA vs GG)**							
Any migraine	5	1442/1880	1.22 (1.05–1.41)	**0.011**	0.63	0	0.72
MO	4	537/1593	1.24 (0.99–1.54)	0.051	0.67	0	
MA	4	717/1593	1.22 (1.00–1.47)	**0.047**	0.76	0	
Higher quality	3	1088/1367	1.21 (1.02–1.44)	**0.028**	0.49	0	
Lower quality	2	354/513	1.23 (0.90–1.68)	0.20	0.29	11	
**Recessive model (AA vs GA + GG)**							
Any migraine	5	1442/1880	1.18 (0.79–1.76)	0.42	0.95	0	0.90
MO	4	537/1593	1.21 (0.68–2.16)	0.52	0.73	0	
MA	4	727/1593	1.20 (0.73–1.98)	0.47	0.99	0	
Higher quality	3	1088/1367	1.25 (0.79–1.99)	0.34	0.84	0	
Lower quality	2	354/513	0.99 (0.45–2.16)	0.97	0.82	0	

*MO, migraine without aura; MA, migraine with aura*.

*p Values <0.05 are in boldface*.

**Figure 3:**
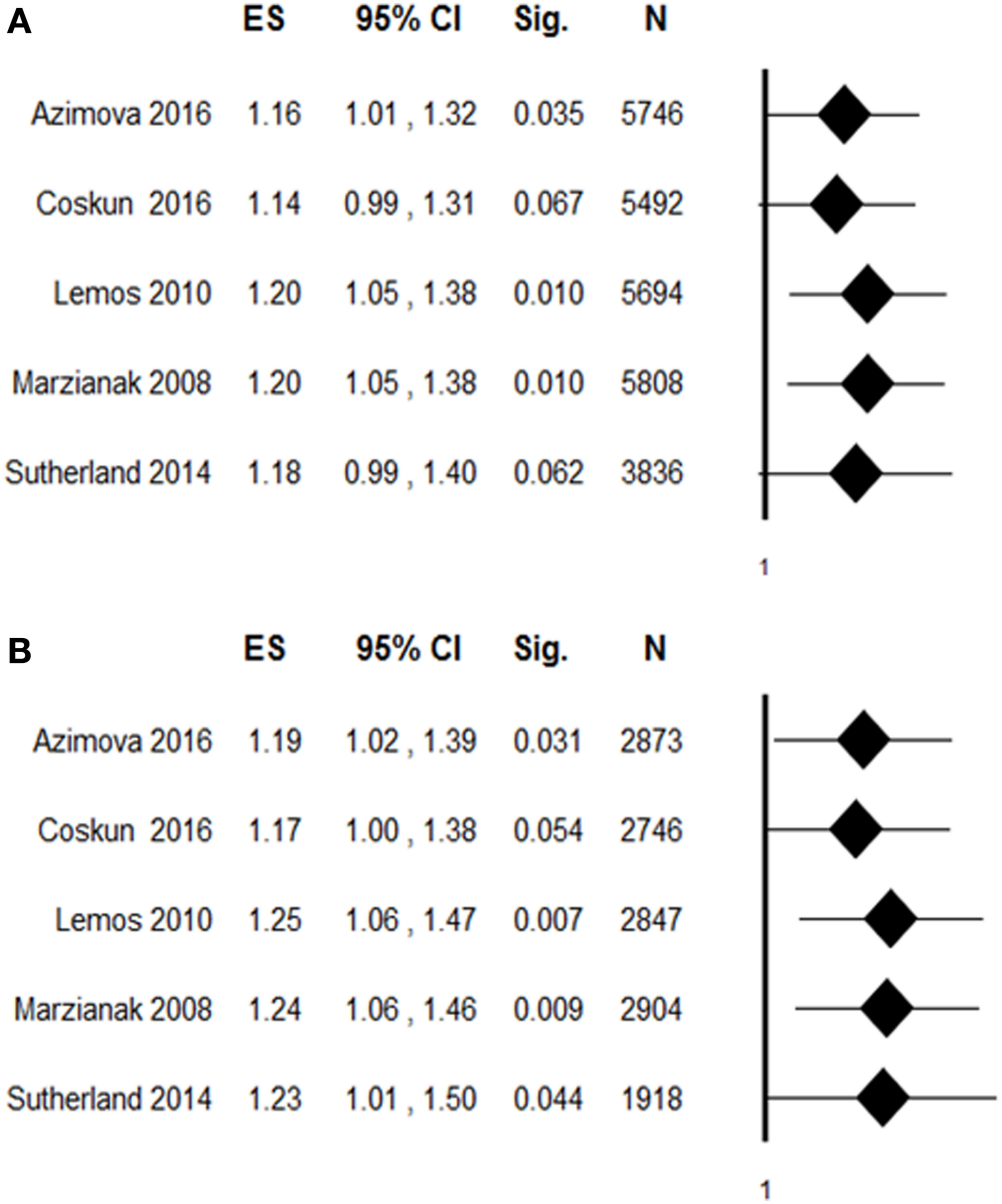
**Leave-one-out sensitivity analysis of the association between rs6265 and migraine susceptibility, by excluding individual studies one at a time and recalculating the pooled odds ratio (OR) estimates for the remaining studies**. **(A)** Allelic contrast of rs6265: A vs G (ref); **(B)** dominant contrast: GA + AA vs GG (ref). ES, effect size (OR); Sig, statistical significance; *N*, total sample.

To evaluate the robustness of overall pooled estimates, we performed a leave-one-out sensitivity analysis by iteratively removing one study at a time and recalculating the summary OR. The results showed that statistical significance of the allelic (Figure 3A) or the dominant (Figure 3B) model of rs6265 was lost when the study of Coskun et al. (21) was excluded from the analysis (dominant OR: 1.17, 95% CI: 1.00–1.38, *p* = 0.054; allelic OR: 1.14, 95% CI: 0.99–1.31, *p* = 0.067). To assess the potential effects of specific study characteristics on the association between rs6265 and migraine susceptibility, we pooled the OR and 95% CI from the subgroups of migraine type and study quality. When stratified by migraine type, a similar trend of association was detected with both MA and MO, but statistically significant association of rs6265 was reached only with the MA subtype in the dominant model (OR: 1.22, 95% CI: 1.00–1.47, *p* = 0.047). When stratified by study quality, statistical significance was reached among studies with higher quality (allelic OR: 1.18, 95% CI: 1.02–1.37, *p* = 0.027; dominant OR: 1.21, 95% CI: 1.02–1.44, *p* = 0.028).

## Discussion

The BDNF rs6265 SNP has been associated with an altered intracellular packaging of the precursor of BDNF and a decreased production of mature BDNF (16), which can lead to a plethora of effects ranging from detrimental molecular, cellular, and brain structural modifications associated with deficits in social and cognitive functions (32). The crucial role of rs6265 on neuronal activity and plasticity mechanisms has been also highlighted in previous meta-analyses of rs6265 that reported an association of this SNP with a range of central nervous system disorders including panic disorder (33), posttraumatic stress disorder (34), idiopathic dystonia (35), and geriatric depression (36).

In the present study, we conducted a systematic review and meta-analysis of published data to quantitatively estimate the impact of BDNF rs6265 on migraine susceptibility. The pooled results have indicated that BDNF rs6265 confers an increased risk for migraine, reaching significant level under the allelic or the dominant model of inheritance. In addition, subgroup analysis based on migraine type shows a statistical significant association with migraine with aura (MA) under the dominant model of rs6265. In this regard, it is noteworthy that the BDNF gene is differentially expressed in the rat after cortical spreading depression (37), the putative underlying mechanism of the migraine aura (38, 39). Despite being the largest study in the meta-analysis and the most populated by MA patients, the study by Sutherland et al. (20) failed to find an association of rs6265 with migraine and, more specifically, with MA. Similarly, no association was detected in all the other studies identified. Our positive findings, in absence of inter-study heterogeneity, suggest that all these studies were underpowered to detect an association of rs6265 with migraine. Yet, on the basis of the effect size suggested in our meta-analysis under the dominant model (OR = 1.22) and assuming an α value of 0.05, a power of 0.80 and a minor allele frequency in controls of 0.169 (365/6644), a sample size of 1,816 case–controls pairs would be required to prospectively detect an association of rs6265 with migraine. A very recent meta-analysis by Cai et al. (40) has reported association of two BDNF gene variants (i.e., rs6265 and rs2049046) with migraine susceptibility (40). While the association of also rs2049046 is not surprising, given its strong linkage disequilibrium with rs6265 (41), it is noteworthy that Cai et al. did not include the study by Azimova et al. (25) in their analysis and they did not perform a subgroup analysis based on migraine type not performed. Thus, we feel that in the present study, we derived a more precise and more comprehensive estimation of the association between rs6265 and migraine.

Our findings suggest that reduced BDNF protein levels in the brain, which are to be expected in carriers of the Met allele of BDNF Val66Met polymorphism (rs6265A), may increase the likelihood of migraine. Intriguingly, positron emission tomography scanning in healthy subjects showed a lower 5-HT1A receptor binding potential in 13 brain regions of interest among carriers of rs6265A allele in comparison with non-carriers (42), a result that is consistent with a model where rs6265 causes less proliferation of serotonin synapses and consequently fewer 5-HT1A receptors. On the other hand, 5-HT1A receptors are implicated in the regulation of central serotoninergic tone, which has long been involved in the pathophysiology of migraine (43), and brainstem changes in 5HT1A receptor availability have been detected during the early stage of migraine attack (44). Based on these previous findings, the results of the present meta-analysis suggest that the Met allele of BDNF rs6265 may contribute to the development of migraine because of its detrimental influence on the central serotoninergic tone. However, further investigation is required to evaluate the impact of rs6265 on the relationship between central serotoninergic system and migraine susceptibility. On the other hand, it is well known that BDNF acts as a pain modulator at multiple levels, including pain processing and drug-seeking behavior. For instance, BDNF rs6265 has been previously reported to influence trigeminal pain-related evoked responses in healthy subjects (45) or to modulate analgesic drug consumption in patients with medication overuse headache (46). Also of note is that the minor T allele of rs6330 of nerve growth factor gene was significantly associated with MA in a Turkish case–control population (21). These results, together with our findings, underline the importance of further research to deepen investigation into the impact of polymorphic variants of genes encoding for neurotrophic factors on susceptibility and clinical features of episodic and chronic migraine.

The limitations of this meta-analysis should not be ignored when interpreting the results. First, the pooled analysis was based on five case–control studies, with a limited numerosity of subjects, who also were mostly Caucasian. In addition, results of leave-one-out sensitivity meta-analysis indicated that the statistical significance of rs6265 was altered when one single study was omitted, thus indicating a lack of robustness of pooled estimates. Thus, caution is needed when interpreting overall and subgroup analyses based on migraine type. Further large studies including populations of different ethnic origin are, therefore, required to provide more conclusive evidence of an association between rs6265 and migraine. Second, migraine is a female-predominant multifactorial disease influenced by complex gene–gene or gene–environment interactions. However, stratified analyses by gender and environmental exposures were precluded owing to insufficient data. Third, the pooled estimates were based on unadjusted data, which might have affected the accuracy of the results. In the future, a collaborative meta-analysis may provide a more precise evaluation of the association between rs6265 and migraine.

In conclusion, our meta-analysis based on five case–control association studies suggested that rs6265 polymorphism of the BDNF gene may confer an increased susceptibility to migraine. However, the impact of gender and ethnicity as well as the investigation of gene-environment interactions should be taken into consideration in future large-scale studies to confirm our findings and to better understand the relationship between the BDNF rs6265 polymorphism and migraine susceptibility.

## Author Contributions

ST performed the critical analysis of the literature and prepared the manuscript. SC has collected and evaluated the data from the literature. She has collaborated to the critical analysis of existing evidence and has collaborated to the preparation of the manuscript. MV has collaborated to the critical analysis of the data and to the preparation of the manuscript. MV has collaborated to the critical analysis of the data and to the revision of the manuscript. CT contributed with the idea of the review, analysis of data, and revision of the manuscript.

## Conflict of Interest Statement

The authors declare that the research was conducted in the absence of any commercial or financial relationships that could be construed as a potential conflict of interest.
